# Case report: A late and isolated presentation of meningoencephalomyelitis uncovers a novel pathogenic variant in the *CIITA* gene

**DOI:** 10.3389/fped.2023.1269396

**Published:** 2023-09-29

**Authors:** Mohammed F. Alosaimi, Muddathir H. Hamad, Muneera J. AlShammari, Dima Z. Jamjoom, Najd S. Musibeeh

**Affiliations:** ^1^ Immunology Research Laboratory, Department of Pediatrics, College of Medicine, King Saud University, Riyadh, Saudi Arabia; ^2^ Allergy and Immunology Unit, Department of Pediatrics, College of Medicine, King Saud University, Riyadh, Saudi Arabia; ^3^ Division of Neurology, Department of Pediatric Neurology, College of Medicine, King Saud University, Riyadh, Saudi Arabia; ^4^ Department of Genetics and Metabolic, College of Medicine, King Saud University, Riyadh, Saudi Arabia; ^5^ Department of Radiology and Medical Imaging, College of Medicine, King Saud University, Riyadh, Saudi Arabia

**Keywords:** type II bare lymphocyte syndrome, CIITA gene, novel mutation, MHC II, genetic disorder, meningoencephalomyelitis, neurologic disease

## Abstract

**Background:**

Bare lymphocyte syndrome type II (BLS II) is a rare form of severe combined immunodeficiency caused by mutations in the *CIITA* gene, which regulates major histocompatibility complex class II (MHC II) expression.

**Objective:**

We report the case of a Saudi boy with a novel mutation in the *CIITA* gene who presented with acute and late meningoencephalomyelitis, resulting in severe neurodevelopmental regression.

**Methods:**

We reviewed the patient's clinical and laboratory data obtained from medical records and performed a literature search on BLS II.

**Results:**

The patient presented with acute meningoencephalomyelitis confirmed by MRI findings and was later found to carry a homozygous pathogenic variant in the *CIITA* gene p.(Leu473Hisfs*15). The patient had no MCH II expression, confirming the genetic diagnosis of autosomal recessive BLS II. Surprisingly, the patient's prior clinical history was unremarkable for significant infections or autoimmunity.

**Conclusions:**

We report a case with a novel *CIITA* gene mutation presenting atypically with a late and isolated severe infection. Isolated severe meningoencephalomyelitis may be a manifestation of primary immunodeficiency.

## Introduction

Bare lymphocyte syndrome type II (BLS II) is a rare inherited form of severe combined immunodeficiency. It was first described in 1978 and is characterized by a lack of major histocompatibility complex class II (MHC II) expression on antigen-presenting cells (1). It manifests with early-onset recurrent infections that mostly affect the respiratory and gastrointestinal systems, resulting in bronchiectasis, malabsorption, and failure to thrive (2). MHC II deficiency is caused by autosomal recessive mutations in genes encoding transcription factors required for MHC II expression. These factors include class II transactivator (*CIITA*), regulatory factor X, ankyrin repeat-containing (*RFXANK*), regulatory factor 5 (*RFX5*), and RFX-associated protein (*RFXAP*) (3).

*CIITA* is encoded by a gene with 27 exons that is mapped to chromosome 16p13.13 and consists of 1,130 amino acids (4). *CIITA* has multiple domains that interact with each other, and it is composed of an amino-terminal acidic domain, proline-, serine-, and threonine-rich (PST) regions, a GTP-binding site, at least one nuclear localization sequence (NLS), and a series of leucine-rich repeats (LRR). A mutation in any of these domains could affect the transcriptional activity of *CIITA* (5).

Here we report the case of a boy who presented with late and isolated acute meningoencephalomyelitis and was later found to have a novel pathogenic mutation in the *CIITA* gene that resulted in complete loss of MHC II expression. His atypical presentation presented a challenge in the diagnosis and management of severe combined immunodeficiency.

## Case report

We present the case of a 3-year-old Saudi male, who was the offspring of an uncomplicated pregnancy and was born at term by spontaneous vaginal delivery with a birth weight of 2.7 kg. He was in a normal state of health until he presented to our emergency department at the age of 2 years with a 9-day history of fever associated with a runny nose, cough, and vomiting. He also had a 3-day history of progressive lethargy and bilateral lower limb weakness, which was more pronounced on the right side. Furthermore, he had a history of profuse, watery diarrhea 2 days before the presentation. There was no history of abnormal jerky movements, drug ingestion, trauma, or contact with sick patients. He was prescribed oral antibiotics for 1 week without improvement.

The patient's development was appropriate for his age, and he had no history of neurological, visual, or cognitive deficits before this presentation. The patient's past medical history was significant only for COVID-19-induced pneumonia at 6 months of age and a history of acute otitis media at 20 months of age. He received all vaccinations up to one year of age without any complications, which included live vaccines like Bacille Clamette-Guerin(BCG), measles, mumps, rubella (MMR), and varicella.

The patient was the first child of a consanguineous (first-degree) Saudi couple. The father was recently diagnosed with neuromyelitis optica (NMO) and was receiving monthly rituximab therapy. There was no family history of primary immunodeficiency or known genetic disease.

On clinical examination, the patient was febrile, lethargic, and hypoactive. He had no facial asymmetry or dysmorphism; his pupils were reactive bilaterally; and he showed no meningeal signs. Tone and strength were more reduced on the right side of his body (2/5) compared to the left side (3/5). Deep tendon reflexes were normal in the upper limbs and left lower limb and diminished in the right lower limb. Gait could not be assessed since the subject could not bear weight. His systemic examination was otherwise unremarkable, and he had no stigmata of neurocutaneous disease.

An urgent brain CT scan was performed and showed no gross evidence of acute brain injury. A full sepsis work-up and serologic investigation were also performed to look for infectious or autoimmune meningoencephalitis, but the findings were unremarkable (results are shown in Table 1).

**Table 1:** Tests performed for the patient.

	Result	Reference range
ANA	1:80 (Fine speckled)	≤1:80
Anti-dsDNA ab	57.54	≤200
ANCA	Negative	<1:20
MPO	1.45	<20
PR3	3.10	<20
Anti SS-A ab	19.54	<20
Anti SS-B ab	2.36	<20
Anti SM ab	2.33	<20
C3	1.450	0.840–1.740
C4	0.248	0.120–0.400
TSH	2.874	0.250–5.0
FT4	14.6	11.5–22.7
Anti TPO ab	124.41	≤100
Anti thyroglobulin Ab	30.42	≤60
Total CK	134	35–232
Vitamin E	11.69	7–21
C/S^a^	Negative	
Varicella IgM	Negative	
Mycoplasma IgM	Negative	
CMV PCR	Negative	
HSV 1 and 2 PCR	Negative	
EBV PCR	Negative	
CSF studies		
Glucose	2.80	2.22–3.85
Protein	0.640	0.150–0.450
WBC _(cells/μl)_	120	≤5
PMN leukocytes _(%)_	5	
Lymphocytes _(%)_	95	
RBC _(cells/μl)_	20	0
Glutamic acid decarboxylase antibodies (GAD65)	3.2	≤10
Aquaporin 4 antibodies	<1:10	<1:10
Myelin oligodendrocyte glycoprotein (MOG abs)	<1:10	<1:10
Anti-ganglioside antibodies	Not detected	Negative
Autoimmune encephalitis serum panel		
Glutamic acid decarboxylase antibodies (GAD65)	51.5	≤10
Aquaporin 4 antibodies	<1:10	<1:10
Myelin oligodendrocyte glycoprotein (MOG abs)	<1:10	<1:10
Anti-ganglioside antibodies	<1:10	Negative

ANA, antinuclear antibody; dsDNA, anti-double stranded DNA antibody; ANCA, antineutrophil cytoplasmic antibody; MPO, myeloperoxidase; PR3, proteinase 3; Anti SS-A ab, anti-SSA (RO) antibody; anti SS-B ab, Anti-SSB (LA) antibody; Anti-SM ab, anti-smith antibody; C3, complement 3; C4, complement 4; TSH, thyroid stimulating hormone; FT4, free thyroxine; Anti-TPO ab, anti-thyroid peroxidase antibody; Anti-Thyroglobulin ab, anti-thyroglobulin antibody; CK, creatine kinase; C/S, culture and sensitivity; CMV, cytomegalovirus; PCR, polymerase chain reaction; HSV, herpes simplex virus; EBV, epstein barr virus; CSF, cerebrospinal fluid; WBC, while blood cell; RBC, red blood cell; PMN, polymorphonuclear.

^a^ C/S included blood, urine, CSF, stool cultures, and a COVID-19 swab.

The patient was admitted to the intensive care unit for neuro-vital monitoring and further management due to progressive weakness. He was started on broad-spectrum antimicrobial and antiviral medications (ceftriaxone, vancomycin, and acyclovir).

**Figure 1:**
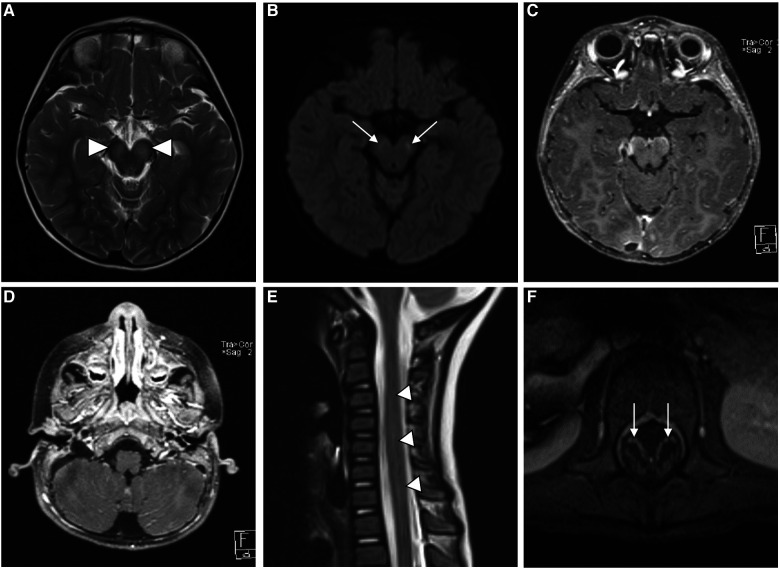
MR imaging of the brain shows bilateral symmetric high signal intensity in the cerebral peduncles (white arrowheads) on T2-weighted images (**A**), with corresponding faint diffusion restriction (white arrows) on diffusion-weighted images (DWI) (**B**) axial postcontrast T1-weighted images (**C** and **D**) show leptomeningeal enhancement (white lines) along the ventral surface of the midbrain and in the cerebellar folia. Sagittal T2 and axial postcontrast T1-weighted images (**E** and **F**) of the spine reveal abnormal hyperintensity of the cervicothoracic spinal cord (white arrow heads in **E**), along with smooth enhancement of the cuada equina nerve roots (white arrow heads in **F**).

Urgent MRI with contrast of the brain and whole spine displayed abnormal T2-weighted-fluid-attenuated inversion recovery (T2-FLAIR) signal intensity and mild diffusion restriction in the midbrain cerebral peduncles bilaterally with leptomeningeal enhancement. Abnormal leptomeningeal enhancement was also seen in the bilateral cerebral hemispheres in the parietal, temporal, and occipital lobes and the pre-chiasmatic segments of the optic nerves. MRI of the spine showed a long segment of abnormal T2 hyperintensity and mild heterogeneous enhancement in the cervical and thoracic spinal cords, along with smooth enhancement along the cauda equina nerve roots (Figure 1). The overall radiologic picture was suggestive of meningoencephalomyelitis. The patient continued to spike a fever for the first 48 hours after admission. However, his fever responded to high-dose intravenous immunoglobulin (IVIG) (1 g/kg of body weight once daily for 2 days). There was no improvement or further deterioration in his neurologic assessment post-IVIG administration. His neuro-ophthalmologic assessment was unremarkable. The patient was discharged home after finishing 3 weeks of antimicrobial therapy. He continued physical therapy with minimal improvement in his lower limb weakness.

**Figure 2:**
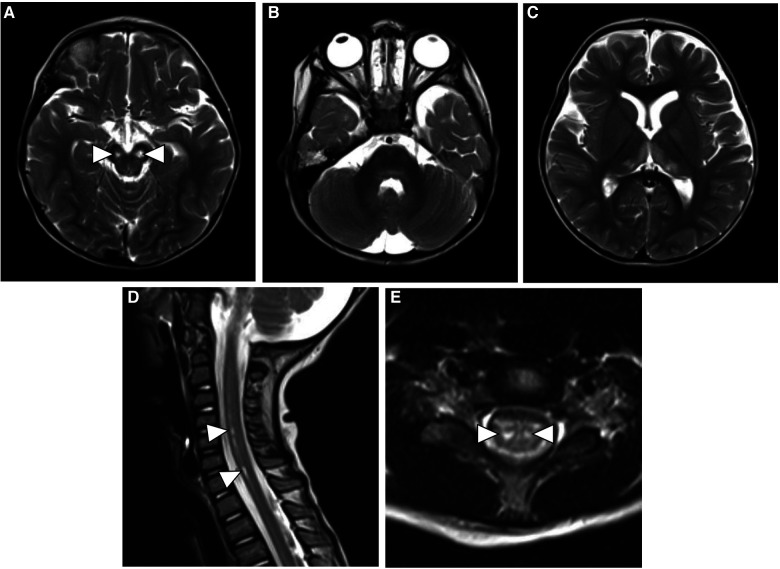
Follow-up axial T2-weighted MR images of the brain (**A**–**C**) show cavitary changes in the cerebral peduncles (white arrowheads) with new areas of hyperintensity in the thalami, globi pallidi and dorsal pons. Sagittal and axial T2-weighted images of the spine (**D** and **E**) show evolution of the previous spinal cord signal abnormality with development of focal areas of myelomalacia in the anterior horn cells (white arrowheads).

At a 3-month follow-up clinic visit, the subject was noted to have developmental regression in gross and fine motor skills with difficulty in phonation. His parents reported repeated episodes of choking, and he eventually required nasogastric tube feeding for oropharyngeal dysphagia. On neurologic examination, he was found to have axial hypotonia and diminished deep tendon reflexes. Follow-up MRIs of the brain and whole spine were performed and showed the progression of signal abnormalities in the cerebral peduncles with the development of areas of cavitation and cystic degeneration. New bilateral areas of abnormal T2-FLAIR hyperintensity developed in the medial thalami, globi pallidi, and dorsal pons. The alterations in the cervical and thoracic spinal cord signals developed into focal areas of myelomalacia and cavitation that predominantly involved the anterior horn cells. The cauda equina nerve root enhancement persisted on follow-up MRI (Figure 2). The possibility of vitamin deficiency or riboflavin transporter deficiency was raised. Therefore, the boy was started on a trial of biotin (10 mg daily), thiamine (300 mg daily), and riboflavin (200 mg daily). However, he failed to respond after 4 months of therapy.

Whole exome sequencing (WES) was ordered clinically to rule out an underlying genetic etiology. It showed that the patient harbored a novel homozygous frameshift variant in the *CIITA* gene (NM_001286402.1:c.1418_1424del:p.Leu473Hisfs*15). The frameshift mutation resulted in a substitution of the leucine residue at 473 to histidine, followed by 15 novel residues and a premature stop codon. It was predicted to disrupt the GTP-binding site domain. Segregation analysis by Sanger sequencing was performed in a clinical lab and confirmed that the patient was homozygous and both parents were heterozygous for the mutation. The mutation led to a complete absence of MHC II expression in B cells (Table 2). The result is consistent with a genetic diagnosis of autosomal recessive bare lymphocyte syndrome, type II, complementation group A.

**Table 2:** Immunologic profile of the patient.

	Result	Reference range (6)
IgA _(g/L)_	**<0.279**	0.140–1.230
IgG _(g/L)_	**0.73**	4.24–10.51
IgM _(g/L)_	**<0.177**	0.480–1.680
IgE _(kU/L)_	<2	0.31–29.5
Lymphocyte _(cells/μl)_	3,200	2,300–5,400
CD3^+^ cells _(cells/μl)_	1,795	1,400–3,700
CD3^+^_(%)_	52.28	56–75
CD4^+^ cells _(cells/μl)_	**405**	700–2,200
CD4^+^_(%)_	12.25	28–47
CD8^+^ cells _(cells/μl)_	1,199	490–1,300
CD8^+^_(%)_	36.27	16–30
CD4^+^/CD8^+^ Ratio	0.34	0.9–3.6
CD16/56^+^ cells _(cells/μl)_	366	130–720
CD16/56^+^_(%)_	10.30	4–17
CD19^+^ cells _(cells/μl)_	1,300	390–1,400
CD19^+^_(%)_	36.55	14–33
CD3^+^ HLA-ABC _(%)_	100	
CD19^+^ HLA-DR DP DQ _(%)_	**0**	

Bold indicates abnormal value.

Based on this finding, the patient was referred to the immunology clinic, and his immunologic workup showed CD4+ T-cell lymphopenia and panhypogammaglobulinemia (Table 2). The patient was started on trimethoprim/sulfamethoxazole prophylaxis and monthly IVIG and referred to a transplant center for possible hematopoietic stem cell transplantation (HSCT).

## Discussion

BLS II is characterized by a lack of MHC II expression, resulting in defective presentation of peptides to CD4^+^ T helper cells. The immunologic consequences of the loss of MHC II are CD4^+^ T-cell lymphopenia, hypogammaglobulinemia, and impaired antibody responses to infection or vaccination (7). Consequently, patients with BLS II suffer from severe immunodeficiency, leading to recurrent infections and ultimately death in early childhood (4).

We reported the case of a 3-year-old male patient who was thriving, with a past medical history remarkable only for COVID-19-induced pneumonia and an episode of acute otitis media at 6 and 20 months of age, respectively. He did not suffer from severe recurrent infections or persistent diarrhea, which are characteristic of BLS II. Instead, the young patient presented with severe acute meningoencephalomyelitis. The clinical severity and radiologic findings raised the question of infectious versus autoimmune causes. The boy's autoimmune panel and infectious workup were inconclusive. The possibility of vitamin deficiency or riboflavin transporter deficiency was raised, but he did not respond to replacement therapy. As a last option to reach a diagnosis, a WES was sent clinically and showed a pathogenic variant in *CIITA.* His immune profile was consistent with BLS II. He had low CD4+ T cells, normal CD8+ T cells, and B cells; reduced levels of IgA, IgM, and IgG; and no MHC II expression on CD19+ cells. Genetic testing showed a novel pathogenic loss-of-function mutation in *CIITA* [c.1418_1424del p.(Leu473Hisfs*15)] that abrogates MHC II expression.

We looked for possible autoimmune encephalitis or vitamin deficiency causes because the patient had a severe case of meningoencephalomyelitis, but no pathogenic organisms were found. Due to this, we tried high-dose IVIG and vitamin (biotin, thiamine, and riboflavin) replacement therapy empirically, but they had no effect on his condition. His autoimmune panels for both serum and CSF came back negative, except for a small increase in serum anti-glutamic acid decarboxylase (GAD65) antibody levels (51.5). This was inconsistent with autoimmune encephalitis, which often has very high serum titers. A working diagnosis of aseptic meningoencephalomyelitis would therefore be the most likely diagnosis given his presentation. Based on a review of reported cases, there have been only 18 reported BLS II patients with a *CIITA* mutation, of which 10 had a missense/nonsense mutation, three had a splicing mutation, one was a regulatory mutation, two had a small deletion mutation, and two had gross deletion mutations [Human Gene Mutation Database (HGMD) 2021] (8–21). A literature review of previously reported patients revealed that none had presented with mainly neurologic manifestations, which constituted a challenge in the diagnosis of our patient (8–21).

BLS II patients have a poor prognosis, and they tend to die early because of severe infections. Treatment options include supportive care with prophylactic therapy with IVIG and antibiotics, but the definitive treatment is HSCT, despite a low success rate of only 60% (22, 23). Our patient was deemed ineligible for HSCT due to his poor neurological condition.

In conclusion, this article reports a case of BLS II presenting late with meningoencephalomyelitis, resulting in severe neurodevelopmental regression without a significant prior infectious history. This finding adds to the wide spectrum of clinical and immunologic phenotypes of such a rare disease and promotes awareness of the disease.

## Data Availability

The raw data supporting the conclusions of this article will be made available by the authors, without undue reservation.
